# A rare case of bowel intussusception due to adenocarcinomatous polyp in a 14 year-old child: case report

**DOI:** 10.1186/s12893-020-00859-9

**Published:** 2020-09-11

**Authors:** Ahmad Sankari Tarabishi, Ziad Aljarad, Baraa Shebli, Ahmad Humam Masri, Rami Anadani, Muhammad Besher Shabouk, Mazen Trissi

**Affiliations:** 1 Faculty of Medicine, University of Aleppo, Aleppo, Syria; 2 Department of Gastroenterology, Faculty of Medicine, Aleppo University Hospital, University of Aleppo, Aleppo, Syria; 3 Department of Neurosurgery, Faculty of Medicine, Aleppo University Hospital, University of Aleppo, Aleppo, Syria; 4 Department of Surgery, Faculty of Medicine, Aleppo University Hospital, University of Aleppo, Aleppo, Syria

**Keywords:** Childhood intussusception, Adenocarcinoma, Jejuno-jejunal invagination, Case report

## Abstract

**Background:**

Intussusception is a form of intestinal obstruction in which a segment of the bowel prolapses into a more distal segment. It is an uncommon condition in children older than 2 years and causes intestinal obstruction. On the contrary of adult intussusception, childhood intussusception does not usually happen on a lead point of a malignant organic lesion.

**Case presentation:**

A 14-year-old male presented with complaints of heavy, bilious emesis and periumbilical colicky pain. Ultrasonography showed a dilated intestinal loop with absent bowel movement. CT scan revealed two masses in the abdomen.

We performed an exploratory laparotomy that revealed invaginated intestines and showed a polyp near the area of interest. Necrotic segments and the polyp were removed and examined pathologically. Pathology showed adenocarcinoma in the polyp.

After surgery, the general condition of the patient was normal and no complications occurred.

**Conclusions:**

Intussusception mainly occurs during infancy and early childhood. Mostly it is an idiopathic ileo-colic invagination. In our case, the patient had a jejuno-jejunal intussusception in his late childhood, and the lead point was an adenocarcinomatous polyp, which is rare in children. Amongst the many types of treatment, we chose surgical resection because of patient’s age.

## Background

Intussusception is a form of intestinal obstruction in which a segment of the bowel prolapses into a more distal segment [1]. It typically occurs from age 6 to 18 months. After 2 years of age, the incidence of intussusception declines. Only 30% of all cases occur in children older than 2 years old [2]. The exact mechanism of intussusception remains obscure in 90% of the cases [3], but an organic lesion may serve as a lead point in initiating its process. Polyps are among the possible precipitating factors [4].

Treatment of intussusception includes enemas, surgical reduction, and surgical resection, however, cases in this age should only be treated with surgical resection [5].

We present in this case a 14-year-old male who was diagnosed with jejuno-jejunal intussusception caused by a polyp that turned out to be an adenocarcinoma.

## Case presentation

A 14-year-old male was admitted to the hospital with complaints of colicky abdominal pain and severe emesis. The patient’s emesis had started 6 months earlier and was mild, but it has worsened during the last month and become more frequent with larger amounts.

The child’s parents had given him over-the-counter antibiotics to treat a suspected gastroenteritis and they had no effect; on the contrary, the symptoms deteriorated bringing them to the hospital. The child had periumbilical colicky pain, nausea, severe emesis, anorexia, weight loss, and he was passing hard stools.

The emesis started reflexive and non-bilious but soon before admission, it turned into bilious emesis. After admission, a Nasogastric Tube (NGT) was inserted and showed bilious emesis followed by fecal emesis, which suggested an intestinal obstruction. The patient did not have any relevant past medical history or drug history or any similar disease in the family.

Physical examination showed a tender, rigid abdomen and no signs of fever. Rectal examination showed a fecal impaction. We evaluated vital signs upon admission: blood pressure: 100/70 mmHg, Pulse: 96 beats/minute.

Lab tests were: (Na^+^: 132 mEq/L) (K^+^: 3 mEq/L) (Glucose: 96 mg/dl) (Creatinine: 0.63 mg/dl) (WBC: 10100 cell/ul) (Hemoglobin: 11.2 g/dl).

We performed ultrasonography to evaluate the suspected intestinal obstruction, which showed some dilated intestinal loops with some fluid congregation. Ultrasonography did not detect any signs of ascites and showed normal liver, spleen and kidneys.

**Fig. 1:**
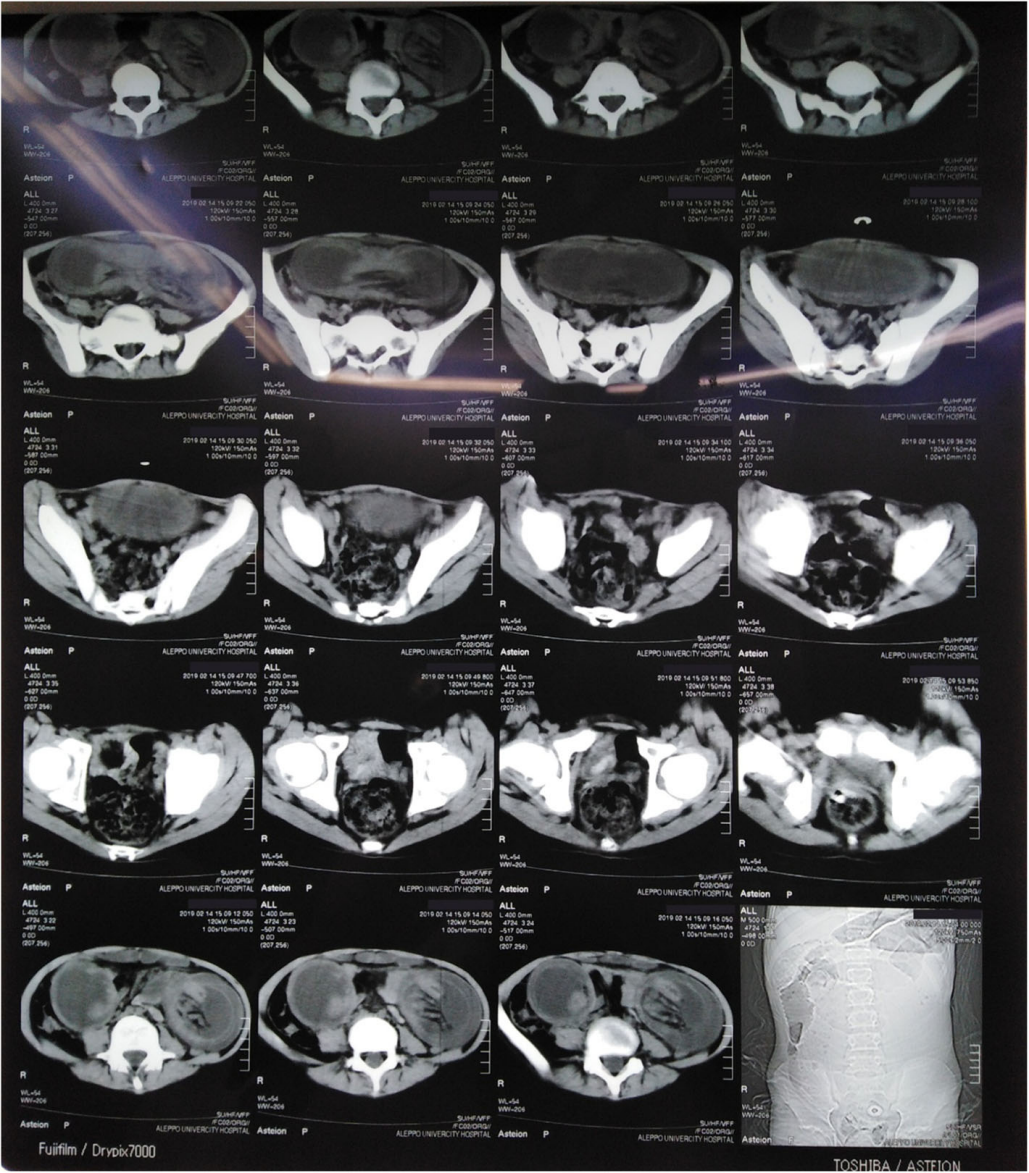
CT scan shows two bilateral abdominal lesions that may indicate dilated, invaginated intestinal loop

CT scan was performed to further evaluate the suspected intestinal obstruction and showed two bilateral abdominal masses that were not suggestive of any specific condition (Fig. 1).

**Fig. 2:**
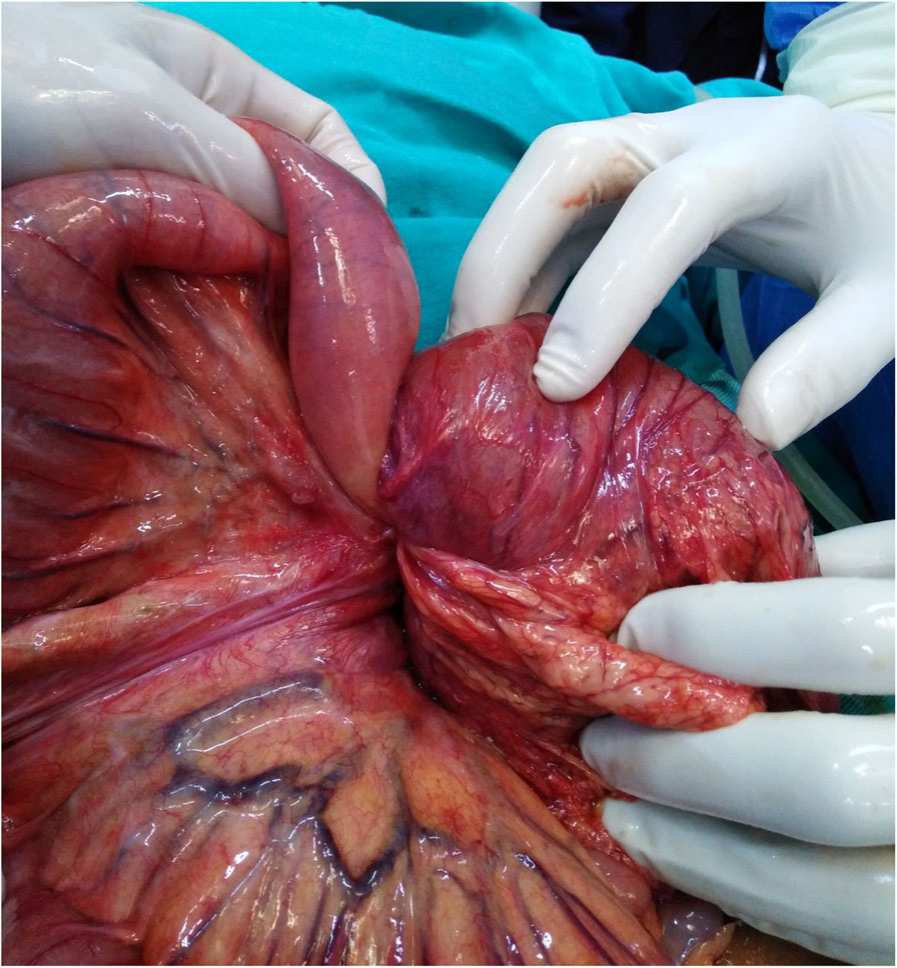
an image during surgery showing signs of necrosis

**Fig. 3:**
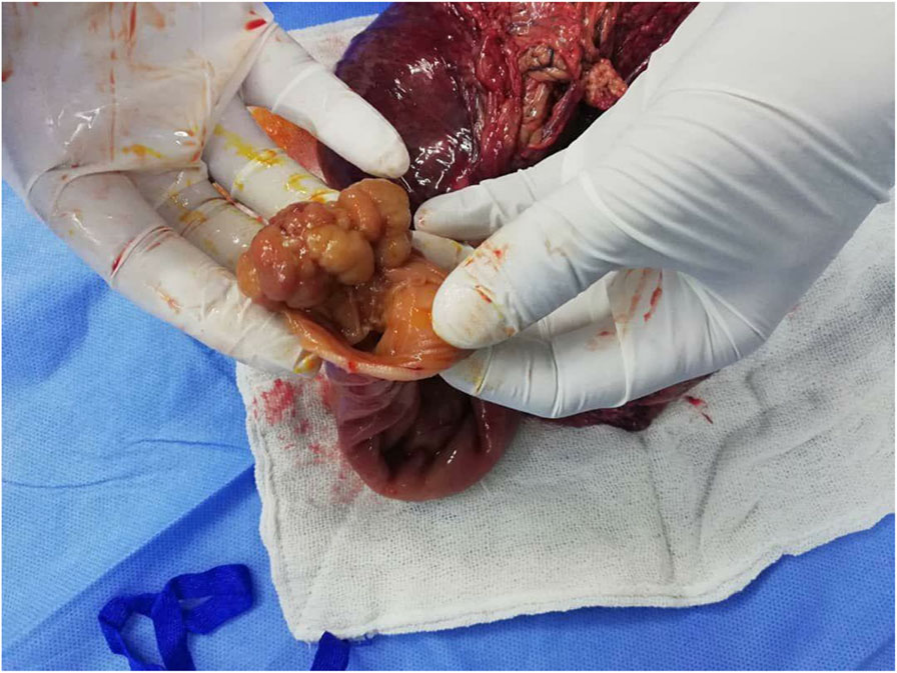
an image during surgery showing an intestinal polyp

The clinical presentations combined with the investigations’ results highly suggested the presence of an intestinal obstruction, which is a surgical emergency. The following exploratory laparotomy showed a jejuno-jejunal intussusception, 70 cm from Treitz ligament. The intussuscepted intestinal segment showed signs of necrosis (Fig. 2) and there was a polyp near the area of intussusception (Fig. 3).

We resected the intussuscepted area, the area of necrosis, the polyp and 34 lymph nodes and sent them to pathological examination. Finally, we performed a primary end-to-end anastomosis.

The patient had a good overall condition after surgery, absent nausea and emesis. We provided oral liquids the second day after surgery.

**Fig. 4:**
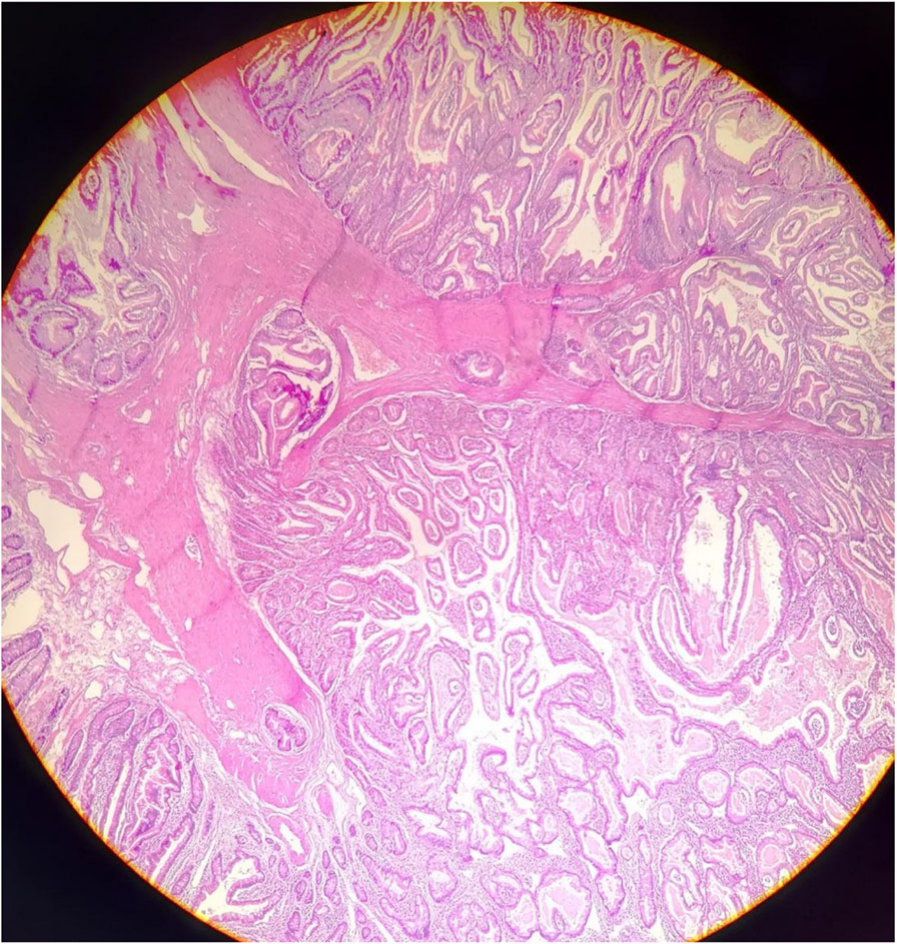
a microscopic image of the biopsy from the resected polyp showing adenocarcinoma

The pathological examination of the polyp reported grade Ι adenocarcinoma with 4.5 cm diameter (Fig. 4). The tumor invaded the intestinal wall and reached the muscularis propria. TNM staging was T = 2, *N* = 0, M = 0 and the tumor required no additional treatment. There was no evidence of metastases in any of the isolated 34 lymph nodes. Unfortunately, we did not genotype the tumor or the patient for the various cancer syndromes because this technology is not available in the province.

Lower gastrointestinal endoscopy after a month showed normal colon and intestines and no polyps. Six months after surgery, we performed a multi-slice CT scan, which was normal.

## Discussion and conclusions

The prevalence of Intussusception in adults is rare, it occurs most commonly in infants aged 5–9 months (67% occur by age 1 year) [6]. The child to adult ratio is more than 20:1 [7]. Our case presented a 14-year-old male with a single polyp in the jejunum. This polyp caused intussusception, which lead to necrosis of the bowels. After surgical resection, the polyp appeared to be malignant.

The histological examination of the polyp that caused the intussusception has revealed an adenocarcinoma. Adenocarcinoma of the bowels is often associated with a number of genetic conditions including: Peutz Jeghers syndrome, familial adenomatous polyposis and Lynch syndrome [8]. The incidence of adenocarcinoma of the large bowel in children is estimated to be 1 in 10 million [8]. Small bowel adenocarcinoma in children is even rarer with only a handful of cases reported.

The intussusception that occurs in the absence of a lead point is classified as primary or idiopathic, whereas in the secondary, a lead point is identified [7, 9]. In our case, the polyp that was found in the jejunum may have been a lead point for the intussusception. Other risk factors that might be associated with intussusception are: proliferation of the lymphatic tissue, intestinal adhesions and infections [1].

Only a few case reports reviewed an intestinal adenocarcinoma diagnosed in children –those cases were caused by specific cancer predisposing syndromes, e.g. Peutz Jeghers syndrome. A study conducted in 1984 showed a 12-year-old girl with primary small bowel adenocarcinoma but there was no polyp and it did not cause an intussusception of the small bowel [10]. Another study reviewed an ileocecal intussusception that appeared in a 50-year-old man and the histologic report confirmed high-grade dysplasia not adenocarcinoma [11].

Regarding prognosis, the univariate predictors for prognosis are: age, severe intestinal symptoms at first diagnosis, T4 of tumor stage, tumor size, relatively late clinical stage, peritoneal metastasis and no chemotherapy. The multivariant predictors are age of more than 60 years old, intestinal obstruction or perforation at first diagnosis, relatively late clinical stage, and no chemotherapy [12].

Early diagnosis of intussusception in adolescent patients is challenging because most cases present with nonspecific symptoms and have a chronic or subacute course [13].

In our case, we decided to perform a surgery-only treatment without adjuvant therapy, because previous studies on small bowel adenocarcinomas showed that adjuvant therapy did not significantly improve overall survival of patients [14], especially with stage I patients [15], and as our patient had stage I adenocarcinoma, systemic adjuvant therapy was omitted.

This decision was taken after discussing the case in a tumor board, where it was recommended that a surgical resection of the adenocarcinoma along with its margins is enough in this case.

In addition, we have completed an oncologic follow-up to assess prognosis of the resected adenocarcinoma and determine whether an adjuvant management is needed. This follow-up, however, was not long enough as the patient did not come back to the hospital due to the hindrances imposed by the local crisis.

The exact cause of developing a gastro-intestinal tract adenocarcinoma in early adolescence is still vague. Studies offer insufficient information about the etiology or risk factors for such cases.

In conclusion, our case is distinct because our 14 year-old patient developed an intussusception, which usually arises in infants, had no signs and no familial record of any cancer predisposing syndrome and exhibited a rare type of intestinal carcinoma. Thus, surgeons treating patients with acute abdomen should always keep in mind that the presenting symptoms of intussusception are non-specific and the underlying causes could be life threatening especially in older patients.

## Data Availability

All data generated or analysed during this study are included in this published article.

## Abbreviations

CT: Computed tomography
NGT: Nasogastric tube
WBC: White blood cell
